# Epidemiological patterns of SARS-CoV-2 reinfections in Espírito Santo, Brazil: A population-based analysis using integrated surveillance and vaccination data

**DOI:** 10.1371/journal.pone.0331771

**Published:** 2025-09-10

**Authors:** Cristiano Soares da Silva Dell’Antonio, Ana Luiza Bierrenbach

**Affiliations:** 1 Instituto de Ensino e Pesquisa, Hospital Sírio-Libanês, São Paulo, São Paulo, Brazil; 2 Secretaria de Estado da Saúde do Espírito Santo, Núcleo Especial de Vigilância Epidemiológica, Instituto Capixaba de Ensino, Pesquisa e Inovação, Vitória, Espírito Santo, Brazil; 3 Precision Data, São Paulo, São Paulo, Brazil; Texas A&M University College Station, UNITED STATES OF AMERICA

## Abstract

**Background:**

Reinfections with SARS-CoV-2 have gained increasing relevance in the context of emerging immune-evasive variants and waning population immunity. Understanding their frequency and distribution is essential to guide public health strategies, particularly in middle-income countries. This study investigates the epidemiological patterns of SARS-CoV-2 reinfections in Espírito Santo, Brazil, using integrated notification and vaccination databases.

**Methods:**

We conducted a retrospective population-based study using state-level surveillance and immunization data from September 2020 to February 2023. Deterministic record linkage was performed to identify reinfections based on four hierarchical definitions, all requiring a minimum 90-day interval between episodes. Reinfection rates were analyzed by demographic, temporal, regional, and vaccination-related variables. Specific contexts such as Indigenous communities and long-term care facilities were also explored.

**Results:**

Reinfection estimates varied widely depending on the definition used—from 0.27% under the strictest criteria to 9.72% under the most inclusive. Reinfections were more common among women and adults aged 30–49 years. The highest proportions occurred during the second and fourth waves, driven by the Gamma and Omicron variants, respectively. Reinfection rates were particularly elevated in Indigenous communities (16.5%) and long-term care facilities (14%), highlighting structural vulnerabilities. In contrast, no increased reinfection risk was observed among incarcerated populations, possibly due to data limitations. Reinfections were frequent among vaccinated individuals, especially those who had received two doses without boosters. However, this likely reflects exposure patterns and time since vaccination, rather than reduced vaccine effectiveness. Brazil’s use of inactivated-virus vaccines, such as CoronaVac, in the early phases may also have influenced susceptibility. Reinfections occurred predominantly after the emergence of Omicron, consistent with findings from national and global studies.

**Conclusions:**

This study shows that the cumulative frequency of reinfections increased over the study period, reaching up to ~10% under the most inclusive definition, while remaining modest under the strictest criteria. These findings reinforce the importance of continued genomic surveillance, timely booster campaigns, and targeted interventions in high-risk environments. The integration of multiple data sources and the use of variable reinfection definitions allowed for a nuanced analysis, offering valuable insights to inform public health planning in Brazil and similar contexts.

## Introduction

The COVID-19 pandemic has significantly impacted global public health. Understanding the dynamics of SARS-CoV-2 infection, including the potential for reinfection, is essential for developing effective disease control strategies.

Reinfection, defined as the occurrence of a second laboratory-confirmed infection (through PCR or rapid antigen testing) in an individual who was previously laboratory-confirmed infected and recovered, became a major concern early in the pandemic. Human coronaviruses, including SARS-CoV-2, are known to cause reinfections, even when humoral immunity from a prior infection is present [1]. While this immunity may not always prevent reinfection, it often helps reduce the severity of subsequent infections by enabling the immune system to mount a more effective response. However, the degree of protection can vary, as immunity tends to wane over time and may be less effective against new viral variants. This highlights the importance of both natural immunity and vaccination in reducing the overall disease burden [2,3]. For the purpose of this study, ‘confirmed’ refers to laboratory confirmation through either RT-PCR or rapid antigen testing for both the initial infection and subsequent reinfection episodes, following established international guidelines [4–6].

Studies suggest that an initial SARS-CoV-2 infection provides considerable protection against subsequent reinfection, although the level of protection can vary across studies. For example, one study estimated a protection rate of 81% against reinfection [7]. However, other studies have reported a range of protection levels, with some showing protection rates between 70% and 90%, depending on specific population characteristics and contextual factors. Lower protection levels are particularly expected in immunocompromised individuals, elderly populations in long-term care facilities, healthcare workers with high viral exposure, and during circulation of immune-evasive variants such as Omicron [8–10]. Combinations of population-level factors, such as low vaccination coverage concurrent with emergence of new variants, can further reduce estimated protection levels at the community level [4,11]. This variability underscores the importance of considering multiple factors, such as waning immunity the emergence of new variants, and the added protection from vaccination after natural infection when assessing overall immunity [12].

The absence of a universally standardized definition of reinfection presents significant sexsechallenges for surveillance and clinical practice. While various health agencies have established criteria – the CDC requires ≥90 days between episodes, the ECDC uses ≥60 days, and some studies apply ≥45 days with additional clinical criteria [5,13,14]. These methodological differences result in substantial variation in reported reinfection rates, ranging from 0.3% to >10% depending on the definition used [4,13,15]. From a clinical management perspective, distinguishing between true reinfection, viral persistence, or relapse is crucial as treatment approaches may differ significantly: reinfections typically require standard isolation and treatment protocols, while persistent infections in immunocompromised patients may necessitate prolonged antiviral therapy or modified isolation periods [11,13]. This ambiguity, coupled with gaps in reliable data collection and reporting, complicates efforts to accurately track reinfections, assess immunity, and guide public health responses [13].

In Brazil, including the state of Espírito Santo, research efforts have focused on understanding the epidemiological aspects of COVID-19, particularly factors associated with hospitalization and mortality. Studies have explored demographic correlations and the impact of the virus on specific population groups, such as children, pregnant women, and healthcare workers [16–21].Despite these efforts, significant gaps remain in our understanding of SARS-CoV-2 reinfection dynamics in Brazil. Specifically, there is limited evidence regarding: (1) population-based reinfection rates using standardized case definitions, (2) risk factors associated with reinfection in diverse demographic groups, (3) the relationship between vaccination status and reinfection risk over time, and (4) reinfection patterns across different pandemic waves and viral variants [13,22]. These evidence gaps are critical for informing public health policies, optimizing vaccination strategies, and developing targeted prevention measures for high-risk populations.

In many countries, including Iran, Mexico, Qatar, South Africa, and several others, studies are addressing these gaps by exploring reinfection rates and the role of immunity in protecting against subsequent infections [13,23–25]. In Israel, research using data from large healthcare providers has revealed a relatively low proportion of reinfection cases [26]. Additionally, case reports and systematic reviews from various regions have documented confirmed instances of reinfection, offering valuable insights into this phenomenon [3,12,27–30].

Our study aims to investigate the COVID-19 reinfection rate in the state of Espírito Santo, Brazil, using notification data from the e-SUS Health Surveillance system, which has been carefully integrated through record linkage. This approach enables the precise identification of reinfection cases by cross-referencing patient records over time, ensuring that the same individual is accurately tracked across multiple infections. By employing this methodology, we can provide robust insights into the reinfection patterns in the state. The findings are expected to deliver valuable guidance for public health policies focused on COVID-19 prevention and control.

## Methods

### Study design and period

This retrospective observational study investigates the COVID-19 reinfection rate in Espírito Santo, Brazil, initially identifying suspected cases based on clinical and epidemiological criteria, subsequently confirmed through laboratory testing (PCR or rapid antigen tests). The study focused exclusively on laboratory-confirmed cases for the final analysis. This approach ensured diagnostic accuracy while maintaining the comprehensive surveillance approach of the e-SUS system, from September 2020 to February 2023. The analysis focuses on different age groups and biological sex (male/female as recorded in official health documentation – Notification Form); cases marked as ‘I’ (ignored) were retained in descriptive totals but excluded from subgroup-specific analyses. We also explored reinfection patterns across different waves of the pandemic, to identify key determinants. Temporal analysis was conducted to evaluate these factors over time, providing insights into reinfection trends throughout the pandemic period.

### Data sources

Two primary databases were used: (1) the e-SUS Health Surveillance system, a comprehensive state-level database that tracks both mild to moderate and severe COVID-19 cases, and (2) the Vacina e Confia immunization system, a state-specific database for monitoring vaccination data. SIVEP-Gripe, a subset of e-SUS, exclusively tracks severe acute respiratory syndrome (SARS) cases and deaths, while e-SUS captures a broader spectrum of cases, making it more suitable for this study. By encompassing both mild/moderate and severe cases, e-SUS VS enables detection of reinfections occurring in outpatient and community settings, ensuring greater representativeness across demographic and geographic strata. Due to limited testing availability during the first three months of the pandemic, notification data from this period were excluded from the primary analysis. In addition to testing constraints, this early period coincided with ongoing modifications to the national surveillance infrastructure to incorporate COVID-19 as a notifiable disease, alongside training of healthcare staff for consistent data entry. These factors contributed to incomplete and heterogeneous data quality. Exclusion of this window follows established epidemiological practice to minimize detection bias during periods of limited surveillance capacity [31,32]. Although we cannot fully rule out the misclassification of early infections, the 90-day minimum interval requirement and the operationalization of systematic testing and contact tracing from September 2020 onward [14] reduce this risk [33]. The analysis begins in September 2020, when mandatory testing for both symptomatic and asymptomatic contacts was introduced [34].

With the end of the global public health emergency and the gradual introduction of pharmacy-based tests and self-tests, there was a significant decrease in the reporting of suspected COVID-19 cases. Mandatory testing for symptomatic and asymptomatic contacts, in place from September 2020, was progressively relaxed starting in mid-2022. From this period onward, case detection increasingly depended on individual initiative, as self-testing became common and positive results were no longer required to be reported. For this reason, the study focuses on the period from September 2020 to February 2023, ensuring greater accuracy and completeness of the information. While COVID-19 cases continued to be reported after March 2023, substantial underreporting occurred due to the transition to endemic surveillance and increased reliance on self-testing. Quantitative bias analysis was not feasible due to lack of reliable parameters to estimate the extent of underreporting in our specific population during this period. This temporal restriction is consistent with similar surveillance studies that focus on periods of systematic case detection [13,35].

Immunization data, which is recorded through the Vacina e Confia system, spans from January 2021 to February 2023 and tracks vaccination coverage throughout the state. Although data is available beyond February 2023, it was only used up to this point to align with the period covered by the notification data.

The Espírito Santo State Health Department operates two dedicated digital platforms: e-SUS Health Surveillance (VS) for mandatory case notifications and Vacina e Confia for immunization records. e-SUS VS was established to replace the SINAN model and was developed in partnership with the Pan American Health Organization (PAHO) to enable centralized, real-time reporting of diseases, conditions, and health events by both public and private providers across all 78 municipalities. Following statewide training of surveillance and primary care professionals in late 2019, the state health department assumed responsibility for consolidating municipal data, updating reporting instruments, providing technical support, and transmitting notifications daily to the Ministry of Health’s Health Surveillance Secretariat (SVS/MS), in line with national reporting deadlines.

Complementing the notification system, Vacina e Confia is Espírito Santo’s official immunization registry for all vaccines administered under the SUS. Developed by the State Department of Health (Sesa), the platform interacts daily with the National Health Data Network (RNDS), the National Immunization Program (PNI) database, and the National Health Registry. Its key features include digital card access via Citizen Access authentication, detailed records of vaccine administration routes and sites, ICD-10 coding for special risk groups, a real-time “Situation Room” dashboard for monitoring coverage and inventory, and centralized management of strategic immunobiologicals across the state’s 78 municipalities.

Importantly, both platforms are independently managed by the state and remained fully operational during the December 2021 cyberattack that disrupted federal health information systems. This ensured uninterrupted access to Espírito Santo’s notification and vaccination records during a period of major national data unavailability.

The databases used for this study do not include unique identifiers consistently across all records. As a result, it was necessary to perform record linkage using nominal information, such as name, date of birth, and other variables.

### Data linkage

The record linkage process was conducted in three distinct stages. In the first stage, applied to the notification database, the objective was to identify records belonging to the same individual, perform deduplication, and define initial infections and reinfections, as well as identify the laboratory tests confirming these events. The second stage, applied within the vaccination database (Vacina e Confia), also involved deduplication to match records to the same individuals and determine how many doses each patient had received and when. The third stage involved merging both databases, integrating the notification and vaccination data. Although the e-SUS database contains fields for recording COVID-19 vaccinations, the data are incomplete, which made it necessary to perform linkage between the notification and vaccination databases.

The datasets were pre-processed for deterministic record linkage, which involved standardizing identification variables. This process included removing special characters, normalizing letter case, managing abbreviations, and cleaning address fields considered non-informative or inconsistent. Corrections were made to patient and mother names, as well as to address information, by fixing common typographical errors, expanding abbreviations with full terms, and addressing invalid or missing values.

Additionally, derived variables were created to aid the linkage process, such as Soundex codes for patient names and addresses. The deterministic linkage process involved applying sequential rules using sets of identification variables to identify duplicate or corresponding records in the notification and vaccination databases. This method, similar to those described in validation studies such as Oliveira et al. [36] and Pacheco et al. [37], was crucial for ensuring data quality and consistency, preventing distortions in the results of the analysis. These rules were applied step-by-step, with each stage based on a specific set of variables, such as CPF (Brazilian individual taxpayer registry), CNS (SUS health card number), patient name, date of birth, and address components. Records containing invalid or missing values were systematically reviewed and cleaned before linkage. This included exclusion of empty or placeholder values, correction of inconsistencies in dates (e.g., inverted digits in birth or vaccination dates), removal of implausible ages (e.g., negative or >125 years), and manual review of cases with conflicting information. No imputation techniques were used; data integrity was preserved by relying on logical corrections and internal consistency checks.

### Linkage quality assessment

The deterministic linkage process between the e-SUS VS (COVID-19 case surveillance) and Vacina e Confia (vaccination) databases was conducted iteratively and incrementally, using multiple combinatorial rules involving identifying variables such as name, mother’s name, date of birth, CPF, CNS, sex, and address [38,39]. The process sought to maximize the accuracy of the matching, even in the face of heterogeneous and incomplete data.

#### Quality assessment.

The quality of the linkage was validated through manual inspection of thousands of paired records, allowing inconsistencies to be identified and corrected. Although formal metrics such as sensitivity, specificity, or false positive rate were not calculated, the procedures adopted followed good methodological practices described in the epidemiological literature.

#### Potential for linkage bias.

Variations in matching rates were observed between population subgroups (babies and children), mainly attributable to the variable completeness of identifying information. Missing or inconsistent data, especially among younger individuals and in areas with lower system coverage, may have led to an underestimation of the matching rate in certain strata.

#### Treatment of conflicting records.

Records with conflicting information between the databases were assessed individually, considering the internal consistency of the data and additional variables available. Doubtful cases were maintained only when there was reasonable evidence that they were the same person.

#### Time alignment.

Dates were standardized and validated to ensure chronological consistency between infection and vaccination. Corrections were applied in cases of obvious typing errors (such as year changes) and records with impossible dates were excluded [40].

#### Limitations.

The main challenge was the lack of consistent unique identifiers between the systems. In a few cases, variables such as CPF and CNS were missing or filled in inconsistently, requiring the use of surrogate variables and textual standardization techniques. This limitation may have resulted in both incorrect pairings and failure to identify records of the same individual.

#### Impact on the study population.

Although a formal sensitivity analysis was not performed, the general patterns of reinfection observed consistent with epidemiological expectations, suggesting that the linkage process did not introduce significant distortions in the composition of the population analyzed.

### Case Definitions

Throughout the study, several case definitions were used to classify individuals:

- **Without Test Result (PCR and TRa):** Patients without any recorded PCR (Polymerase Chain Reaction) or TRa (Rapid Antigen) test result during the study period. This category includes individuals who were not formally tested for COVID-19, with their condition identified without laboratory confirmation.
- **With Test Result (PCR or TRa):** Patients with at least one recorded result from a PCR or TRa test during the study period, regardless of whether the result was positive or negative.
- **Not Infected:** Patients with no positive test results throughout the study period. This definition is based on available surveillance data and does not imply regular or systematic testing. The category includes individuals with negative test results as well as those tested due to clinical suspicion who resulted negative.
- **Infected:** Patients with one or more positive test results (PCR or TRa) during the study period. This definition captures laboratory-confirmed infections regardless of symptoms. While systematic testing for asymptomatic individuals was not universally implemented, Espírito Santo’s surveillance protocol included testing of symptomatic and asymptomatic close contacts, healthcare workers, and individuals in outbreak settings [41].
- **Reinfected:** Patients with multiple positive test results (PCR and/or TRa) separated by at least 90 days, indicating a potential reinfection.
  - **Reinfection Type 1:** Patients with two or more positive PCR tests separated by at least 90 days, with at least one intervening negative PCR result.
  - **Reinfection Type 2:** Patients with two consecutive positive PCR tests separated by at least 90 days, without an intervening negative result.
  - **Reinfection Type 3:** Patients with two or more positive results (PCR or TRa) separated by at least 90 days, with at least one intervening negative test (PCR or TRa).
  - **Reinfection Type 4:** Patients with two consecutive positive results (PCR or TRa) separated by at least 90 days, without requiring an intervening negative test.
- **Not Reinfected:** Patients who did not meet the criteria for reinfection, either due to the time interval between positive tests being less than 90 days or the absence of an intervening negative test where applicable.

### Pandemic waves

To provide a clearer understanding of the epidemiological context, the study identified specific time periods representing distinct pandemic waves. These waves were determined by the researchers based on temporal patterns observed in notification data, accounting for inherent delays in the infection-to-notification process.

Wave identification considered several temporal factors: (1) the median incubation period of SARS-CoV-2 (5.1 days, range 2–14 days) [42]; (2) testing delays, which varied from same-day to several days depending on healthcare system capacity; (3) notification delays from laboratories to surveillance systems; and (4) potential population heterogeneity in testing-seeking behavior.

To account for these delays, wave boundaries were defined using a 14-day moving average of case notifications to smooth short-term fluctuations, and a lag adjustment of 7–14 days was applied to align notification peaks with estimated infection peaks (diagnosis date) [43,44]. Waves were identified as intervals between successive epidemiological nadirs, with each wave characterized by a distinct rising and falling trend in smoothed case counts.

This methodological approach aligns with established epidemiological practices for wave identification during respiratory virus pandemics and provides a robust framework for contextualizing reinfection patterns throughout the study period [44,45].

### Vaccination status

To determine the vaccination status, records from the “Vacina e Confia” system were utilized. Following deduplication and deterministic linkage with the main database, the vaccination doses associated with each patient along the time were identified. For the analysis, the highest dose administered up to 15 days prior to the date of the first infection or reinfection was considered. The 15-day window was chosen to account for the time required for the immune system to generate or boost an effective immune response following vaccination.

### Special residential settings

Patient residence information was also analyzed, where separate variables were created to indicate if the patient resided in a prison, long-term care facility, or indigenous village during their first infection.

### Statistical analysis

Descriptive techniques were employed to analyze the frequencies and proportions of reinfection cases. The reinfection rate was calculated by dividing the number of reinfection cases by the total number of infected cases, then multiplying by 100 to obtain a percentage. This calculation was performed for each of the four reinfection definitions. Analyses were stratified by age group, gender, and across different pandemic waves to explore potential patterns and determinants of reinfection. The analysis was conducted using Stata, version 17.

### Ethical considerations

The study adhered to ethical and regulatory guidelines, ensuring patient privacy and confidentiality. After the record linkage steps, all data were anonymized, and results were presented in an aggregated manner without individual identification. The study was authorized by the Instituto Capixaba de Ensino, Pesquisa e Inovação em Saúde do Espírito Santo and approved by the Ethics Committee of the Instituto de Ensino e Pesquisa do Hospital Sírio-Libanês (CAAE Nº 45986821.9.0000.5461, December 2021), with a waiver for informed consent. In adherence to the STROBE guidelines for observational epidemiological studies, this manuscript addresses the 22-item checklist covering title, abstract, introduction, methods, results, and discussion sections, ensuring transparent and complete reporting.

## Results

**Fig 1:**
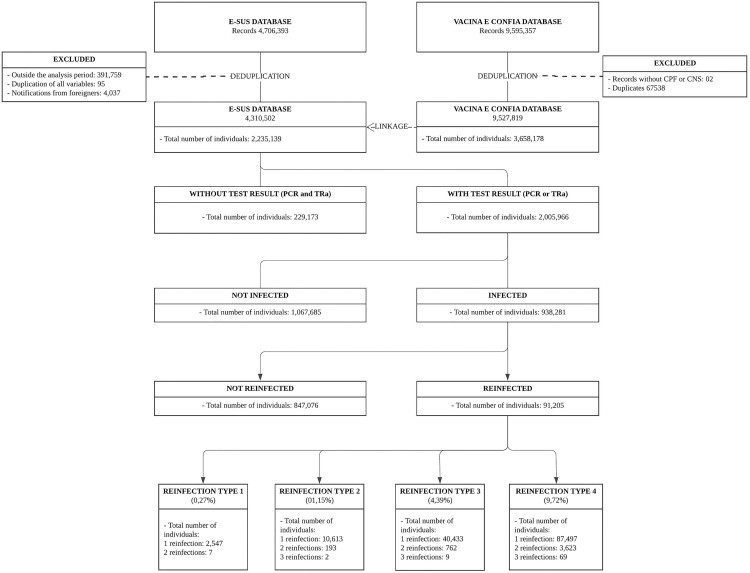
Flowchart of Data Management and Classification of COVID-19 Cases and Reinfections Using the ESUS VS and Vacina e Confia Databases. Espírito Santo, Brazil, September 2020 to February 2023.

Fig 1 presents an overview of the data management process regarding COVID-19 suspected cases and vaccination records. Initially, the e-SUS database contained 4,706,393 records related to suspected COVID-19 infections. Following a thorough deduplication process, this total was reduced to 4,310,502 records, encompassing 2,235,139 distinct individuals. Concurrently, the Vacina e Confia database, which initially held 9,595,357 records, was also deduplicated, resulting in 9,527,819 records representing 3,658,178 individuals. Upon integration of the two datasets, it was observed that 229,173 individuals from e-SUS lacked laboratory confirmation of COVID-19 infection through PCR or rapid antigen (TRa) tests. Among the 2,005,966 individuals with documented test results, 938,281 had at least one positive result (PCR+ or TRa+), while 1,067,685 had a negative result on either test or, when both tests were available, had negative results on both (PCR- and TRa-). Within the infected cohort of 938,281 individuals, reinfection analysis identified 91,205 cases that met the defined criteria for reinfection. These reinfections were further categorized according to four specific rules:

- Reinfection Type 1: Representing 0.27% of the infected group (938,281 individuals), with 2,547 individuals experiencing one reinfection and 7 individuals experiencing two reinfections.
- Reinfection Type 2: 0.15% of the infected group, with 10,613 individuals experiencing one reinfection, 193 with two reinfections, and 2 with three reinfections.
- Reinfection Type 3: 4.39% of the infected group, with 40,433 individuals experiencing one reinfection, 762 with two reinfections, and 9 with three reinfections.
- Reinfection Type 4: 9.72% of the infected group, with 87,497 individuals experiencing one reinfection, 3,623 with two reinfections, and 69 with three reinfections.

The classification criteria become less stringent from Type 1 to Type 4, and as expected, the number of cases identified in each category increases progressively from Type 1 to Type 4.

**Fig 2:**
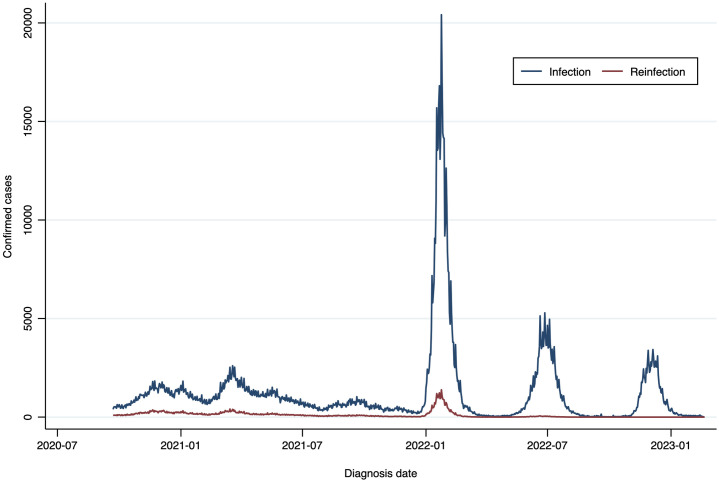
Trends in COVID-19 Infections and Reinfections Over the Study Period. Espírito Santo, Brazil, September 2020 to February 2023.

Fig 2 illustrates the historical series of infections and reinfections from mid-2020 to early 2023. Peaks in the infection series correspond to distinct waves of COVID-19. The pattern suggests six waves: a small rise in early 2021, peaking in January; a larger wave in mid-2021, peaking in July; a notable peak in late 2021, reaching its highest point in October; the highest peak in early 2022, likely Omicron-related, with a peak in January; another wave in mid-2022, peaking in July; and a final, smaller wave at the end of 2022 into early 2023, peaking in December. Reinfections follow a similar pattern, with lower counts, increasing notably during the Omicron wave. In the last three waves, clear nadirs between peaks make it easier to distinguish each wave. This clarity contrasts with the earlier waves, where higher baseline levels and more gradual declines made it difficult to separate individual waves.

Table 1 presents COVID-19 reinfection rates across various characteristics. Reinfections were predominantly Type 4, accounting for 9.72% of the total infected population. Females exhibited higher reinfection rates across all types, with Type 4 reaching 10.99% compared to 8.09% in males. Reinfection rates increased with age, peaking in the 30–39 (11.46%) and 40–49 (11.67%) age groups, while children aged 0–9 had the lowest rates. Regionally, Type 4 reinfections were highest in the Central region (10.64%), followed closely by the Metropolitan area (9.99%). Peaks occurred during the second (11.87%) and fourth (11.22%) COVID-19 waves, while later waves showed minimal reinfections. There were notably high Type 4 reinfection rates in long-stay institutions (14.04%) and Indigenous villages (16.49%). Regarding vaccination status, individuals with additional doses and boosters had higher proportions of Type 4 reinfections, reaching 15.65% for those with additional doses and 13.68% for individuals with one booster.

**Table 1:** Profile COVID-19 Infections and Reinfections. Espírito Santo, Brazil, September 2020 to February 2023.

		INFECTION	TYPE 1		TYPE 2		TYPE 3		TYPE 4	
N		938.281	2.554	0,27%	10.808	1,15%	41.204	4,39%	91.189	9,72%
Sex* ***	M	410.855	927	0,23%	4.146	1,01%	13.884	3,38%	33.244	8,09%
	F	527.250	1.627	0,31%	6.660	1,26%	27.318	5,18%	57.939	10,99%
	I	176	0	0,00%	2	1,14%	2	1,14%	6	3,41%
Age Range*	0 - 04	21.210	7	0,03%	56	0,26%	223	1,05%	594	2,80%
	05 - 09	24.088	10	0,04%	75	0,31%	268	1,11%	846	3,51%
	10 - 19	81.930	111	0,14%	559	0,68%	2.049	2,50%	5.079	6,20%
	20 - 29	164.430	587	0,36%	2.305	1,40%	8.118	4,94%	17.320	10,53%
	30 −39	200.403	816	0,41%	3.164	1,58%	10.924	5,45%	22.976	11,46%
	40 - 49	172.025	558	0,32%	2.274	1,32%	9.316	5,42%	20.073	11,67%
	50 - 59	128.229	285	0,22%	1.365	1,06%	5.952	4,64%	13.431	10,47%
	60 - 69	85.077	131	0,15%	675	0,79%	2.982	3,51%	7.117	8,37%
	70 - 79	41.027	35	0,09%	245	0,60%	1.023	2,49%	2.715	6,62%
	80 - 89	16.300	13	0,08%	76	0,47%	306	1,88%	883	5,42%
	90 +	3.562	1	0,03%	14	0,39%	43	1,21%	155	4,35%
Region of residence *	Central	141.088	48	0,03%	285	0,20%	6.502	4,61%	15.005	10,64%
	Metropolitan	542.813	2.340	0,43%	9.440	1,74%	25.624	4,72%	54.203	9,99%
	North	94.720	53	0,06%	315	0,33%	3.306	3,49%	7.723	8,15%
	South	153.012	113	0,07%	761	0,50%	5.728	3,74%	14.123	9,23%
Wave*	1	47.081	267	0,57%	955	2,03%	1.786	3,79%	3.115	6,62%
	2	213.536	1.350	0,63%	4.888	2,29%	13.389	6,27%	25.340	11,87%
	3	361.319	811	0,22%	4.077	1,13%	15.755	4,36%	35.780	9,90%
	4	222.752	119	0,05%	810	0,36%	9.765	4,38%	24.988	11,22%
	5	67.544	7	0,01%	77	0,11%	509	0,75%	1.965	2,91%
	6	26.049	0	0,00%	1	0,00%	0	0,00%	1	0,00%
Special residential settings*	Prison	967	0	0,00%	6	0,62%	16	1,65%	51	5,27%
	Long-stay institutions for the elderly	520	1	0,19%	7	1,35%	64	12,31%	73	14,04%
	Indigenous village	552	2	0,36%	4	0,72%	43	7,79%	91	16,49%
Anti-COVID-19 vaccination dose**	1st Dose	22461	63	0,28%	350	1,56%	679	3,02%	2099	9,35%
	2nd Dose	115156	631	0,55%	2596	2,25%	6954	6,04%	18486	16,05%
	^+^Unique	5348	19	0,36%	95	1,78%	274	5,12%	787	14,72%
	Additional Dose	5603	30	0,54%	99	1,77%	409	7,30%	877	15,65%
	One booster	117348	444	0,38%	1544	1,32%	8049	6,86%	16058	13,68%
	More than one booster	111399	140	0,13%	465	0,42%	5765	5,18%	8675	7,79%

* By date of diagnosis of the first infection; ** Maximum dose recorded up to 15 days before the date of first infection (Infection) or reinfection (Type 1,2,3,4); *** Biological sex – M: Male, F: Female and I: Ignored; + Single dose of the Jansen vaccine.

## Discussion

This study provides a comprehensive analysis of SARS-CoV-2 reinfections in Espírito Santo, Brazil, revealing key demographic, regional, and temporal patterns. The proportion of reinfected individuals varied considerably depending on the criteria applied, ranging from 0.27% under the strictest definition to 9.72% using the most inclusive approach. Higher reinfection rates were observed among women and adults aged 30–49 years, as well as in specific regions—particularly Greater Vitória (the metropolitan area) and certain vulnerable communities, such as Indigenous villages. Notably, reinfections were concentrated during the state’s second and fourth pandemic waves, corresponding to the circulation of the Gamma and Omicron variants, respectively. Interestingly, among vaccinated individuals, those with a complete primary series but no booster dose appeared to have a higher reinfection rate within the vaccinated group. In the sections that follow, we critically examine these findings in light of national and international literature, highlighting areas of convergence and divergence in reinfection rates and methodological approaches, as well as the methodological advances and local relevance of our data.

### Definitions and their implications

This study applied four hierarchical definitions of SARS-CoV-2 reinfection, requiring ≥90-day intervals between positive tests, with stricter criteria also demanding an intermediate negative result. Reinfection estimates ranged from 0.27% to 9.72% depending on the definition, underscoring how methodological choices directly affect observed rates. This variation mirrors findings by Chemaitelly et al. [4], who showed that stricter definitions, such as the CDC’s ≥90-day threshold, may underestimate reinfection. Some agencies, like the ECDC, use ≥ 60 days instead [7].

Long intervals may miss reinfections occurring within shorter windows, especially with more transmissible or immune-evasive variants. Conversely, short intervals risk capturing prolonged viral shedding as reinfection. Yamasaki and Moi [13] argue for combining clinical, temporal, and genomic criteria—where available—for more accurate classification.

Early in the pandemic, reinfection case definitions relied on genomic confirmation. However, as global case numbers surged, routine sequencing became impractical, prompting surveillance systems worldwide to adopt time-based criteria. In this context, our study employs a hierarchical classification system that, although not aligned with formal standards, offers a structured framework to assess how varying definitional stringency influences reinfection detection.

While the reinfection types defined in our study are not mutually exclusive—a recognized limitation—Type 4 serves as the primary outcome measure, capturing the broadest range of possible reinfections. Types 1–3, in turn, act as sensitivity analyses, reflecting progressively stricter criteria that enhance specificity at the expense of sensitivity. This strategy provides insight into how conservative versus inclusive definitions can shape reinfection estimates, enabling more meaningful comparisons across studies employing different methodologies.

Our findings support the growing consensus in the literature that reinfection rates must be interpreted in light of the definitions applied [13,46], especially during periods of high transmission or the emergence of new variants. Moving forward, surveillance systems should adopt a single, standardized case definition tailored to local testing capacity and public health goals, while relying on sensitivity analyses to evaluate robustness—rather than presenting multiple overlapping categories as equivalent outcome measures.

While early in the pandemic the definition of a reinfection required genomic confirmation, the scale of global infections quickly made sequencing infeasible. Time-based definitions were then adopted by surveillance systems worldwide. By applying multiple definitions in parallel, our study offers a comparative perspective and shows that the more inclusive definitions yield reinfection rates consistent with national data, such as ~8% reported by Fonseca et al. [47]. In contrast, strict definitions captured only a small fraction of events (~0.3%).

These findings reinforce calls in the literature to interpret reinfection estimates in light of the definitions used, especially during periods of intense viral circulation or variant emergence.

### Gender distribution

Reinfection rates in Espírito Santo were higher among women across all methodological definitions. Women accounted for 527,250 (56.2%) of initial infections but represented a disproportionate majority of reinfection cases across all definitions, ranging from 61.6% to 66.3%. Under the broadest definition (Type 4), 57,939 of 527,250 infected women experienced reinfection (10.99%), compared to 33,244 of 410,855 infected men (8.09%). This pattern of female predominance in reinfections aligns with findings from the UK, where women represented 67% of reinfection cases despite accounting for only 53% of initial infections [7], and with a systematic review that identified higher reinfection risk in women [48].

This may reflect greater occupational exposure, as women constitute a large share of the health and education workforce—sectors with high transmission risk [3,17]. Biological factors have also been proposed: although men often have more severe primary infections, women may mount a stronger short-term immune response that wanes more quickly, increasing susceptibility to reinfection over time [28,49].

Additionally, health-seeking behavior differences may have contributed: women are more likely to seek medical care and adhere to testing recommendations, particularly after the relaxation of mandatory testing. This behavioral factor may have amplified reinfection detection among women in Espírito Santo. However, not all studies show gender differences—analyses from Qatar and Israel found similar reinfection rates between sexes [26,50]. In Brazil, both our findings and those of Fonseca et al. [47] suggest a modest female predominance, potentially shaped by exposure patterns, health-seeking behavior, and reporting practices. These results highlight the value of tailoring preventive strategies to occupational and social contexts, particularly in predominantly female environments like schools and health facilities.

### Age distribution

Reinfection age-stratified risks followed a non-linear pattern, with adults aged 30–49 showing the highest reinfection rates (≈11–12% under the broadest definition), while children and older adults demonstrated lower proportions. This distribution pattern reflects complex interactions between exposure risk, vaccination coverage, and demographic behaviors.

Analysis of vaccination status by age stratification revealed a pronounced gradient across age groups. Older adults (60 + years) exhibited significantly higher proportions of multiple booster doses among reinfection cases (≈51% under the broadest definition) compared to all younger age groups, where proportions ranged from 4.0% in 10–19 years to 24.3% in 50–59 years (Supplementary S1 Table). This gradient likely reflects vaccination policy prioritization of elderly populations, age-related immunosenescence requiring additional doses, and greater adherence to booster campaigns among older adults [23].

Notably, cases with only first-dose vaccination remained consistently low across all age groups (4–7%), which may reflect the temporal progression of vaccination coverage during the study period, with most individuals having received additional doses by the time reinfections occurred. These findings align with international evidence suggesting that working-age adults face greater exposure due to occupational and social mobility patterns.

Some studies, like Mensah et al. [7], reported older age among reinfection cases—possibly reflecting delayed exposure or survival through earlier waves. In Espírito Santo, our findings may reflect both higher occupational and social exposure among working-age adults, as well as a survivorship bias, in which individuals who survived earlier waves remained in the population at risk for reinfection during subsequent waves. This survivorship effect is particularly relevant in longitudinal cohort studies, where the population available for follow-up consists primarily of those who survived previous infection episodes, potentially creating a selection bias toward individuals with certain immune or behavioral characteristics.

Although immunosenescence in the elderly may theoretically increase susceptibility to reinfection, greater vaccine coverage and precautionary behaviors in this population likely mitigated this risk [51] in our study. Conversely, children demonstrated lower reinfection rates, possibly due to reduced testing frequency and different immune response patterns compared to adults.

Our results demonstrate consistency with Latin American epidemiological data [25] and cohort studies among essential workers [3], reinforcing the critical need to prioritize booster campaigns and preventive measures for working-age adults while maintaining robust protection strategies for age extremes.

### Regional distribution

Marked geographic differences were observed. While Greater Vitória registered the highest number of reinfections in absolute terms—consistent with its large population and intense transmission—the Central region of the state showed one of the highest proportional reinfection rates (~10–11%), suggesting that smaller municipalities also experienced multiple waves affecting the same individuals.

Reinfection rates were especially elevated in certain vulnerable communities, such as Indigenous villages, where they reached 16%. Similar patterns have been linked to limited isolation capacity and lower pre-existing immunity in remote populations [20].

High reinfection rates in populous areas reflect repeated epidemic waves, as seen in national data showing that the most populated states of São Paulo, Minas Gerais, and Rio de Janeiro concentrating most reinfections [47]. At the same time, localized surges in interior areas may result from household clusters or mass gatherings.

Although international literature rarely presents subnational detail, studies in South Africa and Mexico also found reinfection rates varied by region, reflecting differences in variant spread and wave intensity [23,25].

By highlighting these disparities within a single state, our findings emphasize the need for region-specific policies—such as enhanced surveillance in rural hotspots and tailored vaccination in underprotected areas, including Indigenous communities.

### Pandemic waves and viral lineages

Our findings confirm that reinfections in Espírito Santo peaked during the second and fourth waves—periods dominated by the Gamma and Omicron variants, respectively. The second wave saw the introduction of Gamma, which partially evaded immunity from prior infections [51,52]. The fourth wave, driven by Omicron, marked an unprecedented spike in reinfections, with over 11% of cases involving individuals previously infected.

This pattern mirrors global data. In South Africa, Pulliam et al. [23] were among the first to report a sharp increase in reinfection risk during the Omicron wave. A meta-analysis by Chen et al. [30] estimated reinfection incidence of 3–5% during Omicron’s spread—far higher than during Beta or Delta waves.

In our data, reinfections remained rare in 2020, rose modestly during Gamma’s emergence, and surged during Omicron. Notably, Delta did not produce a marked reinfection increase, likely due to more limited immune escape compared to Gamma and Omicron [50,53]. Also, by late 2021, over a year had passed since initial infections, and waning immunity may have left many susceptible even without major antigenic shifts [29].

These temporal patterns suggest that reinfections may be influenced by both viral evolution and potentially waning immunity, though our observational data cannot definitively establish causal relationships between these factors. Local trends in Espírito Santo closely tracked lineage replacement, reinforcing the value of real-time genomic surveillance to anticipate reinfection waves and guide timely booster campaigns.

### Vaccination status

Reinfections in Espírito Santo occurred across all vaccination groups, including individuals with booster doses. Among the vaccinated, those with only the primary two-dose series appeared to have higher reinfection rates than even some partially or unvaccinated groups. However, this pattern should not be interpreted as evidence of vaccine failure, as our study design does not allow for direct assessment of vaccine efficacy —rather, it reflects important timing and exposure biases.

By the time the major reinfection wave (Omicron) struck, a large share of the population had already been vaccinated. Thus, most reinfections occurred in people who had received at least two doses, simply because they constituted the majority. Additionally, individuals prioritized for early vaccination—such as healthcare workers and older adults—were also those at higher risk of repeated viral exposure or reduced immune responses due to age. These groups were more likely to face reinfection despite being vaccinated, not because of vaccine inefficacy, but due to their heightened vulnerability.

Furthermore, the reinfection risk increased as vaccine-induced immunity waned. Many individuals infected during Omicron had completed their primary series several months earlier and had not yet received a booster. International studies confirm that protection against infection declines over time, particularly without updated boosters, even as protection against severe disease remains high [4,10].

Brazil’s early immunization campaign also relied heavily on traditional platforms such as CoronaVac, which, while essential for coverage expansion, showed lower and less well-characterized effectiveness against immune-evasive variants compared to mRNA vaccine [54]. This context may have further influenced reinfection dynamics, especially in the absence of timely boosters.

Even so, global evidence supports the effectiveness of hybrid immunity and booster doses in reducing reinfection risk [12,25]. Our data suggest that the reinfections observed—particularly during the Omicron wave—were driven by a convergence of waning immunity, viral evolution, and sustained exposure rather than indicating reduced vaccine performance, though our study design does not permit direct evaluation of vaccine effectiveness.

These findings reinforce the critical importance of timely booster campaigns, especially in high-risk and high-exposure populations, and the need for continued genomic and epidemiologic surveillance to anticipate new waves of transmission.

### Reinfection rates in specific settings

This study identified elevated reinfection rates in certain closed and vulnerable settings. In long-term care facilities, about 14% of residents experienced reinfection—well above the population average—likely due to advanced age, weaker immune responses, and prolonged exposure [55].

Among Indigenous communities in Espírito Santo, reinfections reached 16.5%, the highest rate observed. Successive outbreaks in these communities may reflect socio-environmental factors such as communal housing, limited isolation capacity, and delayed early exposure to the virus [20].

In contrast, reinfection rates among incarcerated individuals did not appear elevated in our data. However, this finding must be interpreted with caution. Due to data limitations, we could not distinguish between different types of incarceration facilities—such as short-term jails versus long-term prisons—which may differ in exposure and outbreak control. Moreover, underreporting is possible due to barriers to testing access, hospital transfers, and the frequent use of non-carceral addresses in notification records. These factors may have led to misclassification or underestimation of reinfection rates in this population.

While our study did not present specific reinfection estimates for healthcare workers, existing literature documents high incidence in this group, especially during the Omicron wave [3,17].

Overall, the findings highlight the need for tailored interventions in institutional and collective settings—including vaccination, testing access, and early outbreak response—particularly among long-term care residents and Indigenous populations. For correctional facilities, more granular data would be necessary to clarify reinfection risks and guide specific protective strategies.

### Comparison with other reinfection studies

Our findings align with global and national evidence showing that SARS-CoV-2 reinfections became more frequent over time, particularly during the Omicron period. Early studies, such as Hall et al. [46] in the UK and Abu-Raddad et al. [50] in Qatar, reported low reinfection rates (<1%) during the pre-Omicron phase—consistent with our strictest criterion (0.27%).

Later studies showed increasing reinfection rates. Chen et al. [30] estimated a global prevalence of 3–5% during Omicron. Our broader criterion (~9.7%) may appear higher, but it includes antigen tests and does not require a negative intermediate test—unlike stricter definitions used in some international studies [13,56]. When aligned methodologically, our estimates converge with international findings.

In Brazil, Fonseca et al. [47] found a reinfection rate of ~3.6% (≥60-day interval) by mid-2022, rising to ~8% during Omicron. These figures closely match our data from Espírito Santo, despite different sources (notifications vs. private labs), underscoring consistency. Both studies reported that over 90% of reinfections occurred after late 2021.

Estimates from high-risk groups also align: Cegolon et al. [3] reported ~13% reinfection in Italian healthcare workers, and Michael et al. [55] observed ~18% among residents in long-term care facilities. Our 10% overall rate is consistent with this range, considering population risk heterogeneity.

Importantly, some international studies count multiple reinfections per person; our analysis considered only the first. Nonetheless, multiple reinfections remained rare in our dataset, reinforcing global findings.

In terms of risk factors, our results corroborate prior studies highlighting exposure (e.g., healthcare, education), lack of boosters, and variant emergence as key drivers [12,57]. Additionally, our study offers novel insights by detailing subnational and vulnerable group patterns—particularly in Indigenous communities, which are often absent from international analyses.

## Strengths and limitations

This study has several strengths. By leveraging a large, population-based dataset that includes both mild and severe cases and integrating case notifications with state-level immunization records, we were able to capture a broader and more representative picture of reinfections than studies relying on limited clinical or laboratory cohorts. The deterministic record linkage process was rigorous, with extensive data cleaning, standardization, and manual review of samples to minimize errors. Although applying multiple reinfection definitions does not in itself constitute a novel technical method, our hierarchical framework offers an analytical tool to demonstrate explicitly how different case‐definition choices can substantially alter reinfection estimates. We have therefore reframed Definitions 1–3 as sensitivity analyses and Definition 4 as our primary outcome, clarifying that each serves a distinct purpose. We further encourage future validation studies—linking detailed clinical or sequencing data to notification records—to formally quantify the sensitivity and specificity of each criterion and guide selection of the most appropriate definition for specific research or surveillance objectives. Finally, the inclusion of regional and subgroup analyses, such as Indigenous communities and long-term care settings, adds granularity often lacking in broader national or international reports.

Nevertheless, some limitations should be noted. First, the use of secondary data may be subject to underreporting or delayed notifications, particularly for mild or asymptomatic cases. Second, despite robust linkage procedures, matching errors cannot be entirely excluded—though results were consistent with external benchmarks, suggesting minimal impact on validity. Third, reinfection definitions did not include genomic confirmation, meaning some cases could reflect prolonged viral shedding; the ≥ 90-day interval helps mitigate this, as supported by prior studies [4].

Data on vaccination were derived from Espírito Santo’s state system (“Vacina e Confia”), which remained intact following the federal cyberattack in late 2021. While this improved completeness, small discrepancies with national-level studies using federal datasets may arise. Additionally, variant assignment was based on epidemiological week reports, not individual sequencing, limiting precision in identifying which variant caused a given reinfection.

Given the passive surveillance framework, asymptomatic and mild infections may be underdetected, potentially biasing reinfection rates. Future studies should implement active surveillance protocols—such as periodic community testing and cohort monitoring—to capture subclinical reinfections and refine incidence estimates.

Finally, behavioral and social variables—such as masking, testing behavior, or household exposure—were not captured, potentially introducing unmeasured confounding. For example, cautious individuals might be both less exposed and less likely to test again when asymptomatic, potentially biasing associations. Despite these caveats, the consistency of our results with national and international evidence reinforces their robustness.

## Conclusion

This study presented a comprehensive analysis of SARS-CoV-2 reinfection rates in the state of Espírito Santo, Brazil, highlighting the demographic, regional, and temporal factors associated with this phenomenon. By integrating state-level case notification and vaccination data through robust record linkage, we were able to explore the dynamics of reinfections across different pandemic waves and the emergence of new variants. Reinfection rates ranged from approximately 0.3% (under the strictest criteria) to nearly 10% (under the most inclusive), depending on the definitions applied. Higher reinfection incidence was observed among women and adults aged 30–49 years, as well as in certain regions and specific contexts, such as metropolitan areas and Indigenous communities. Additionally, vaccination status played a role: reinfections occurred even among individuals with a two-dose primary series, suggesting that factors such as waning immunity, the emergence of highly transmissible variants, and uneven vaccine coverage contributed to the observed patterns. From a public health perspective, our findings underscore the need for tailored response strategies at both local and national levels. Vaccination policies should prioritize the groups and regions most affected by reinfections, and continuous genomic surveillance remains crucial for promptly identifying emerging variants and guiding timely interventions. Special attention should also be directed toward vulnerable populations, such as residents of long-term care facilities and Indigenous communities, to ensure equitable protection and reduce health disparities. Despite the inherent limitations of using secondary data and varying reinfection definitions, the alignment of our results with global literature and the rigorous methodology employed lend robustness to our conclusions. In summary, these findings provide valuable scientific evidence to inform public policy aimed at mitigating the impact of COVID-19, particularly in light of emerging variants and potential future pandemic scenarios.

## Supporting information

S1 Table COVID-19 reinfection cases stratified by age group and vaccination status according to four methodological definitions, Espírito Santo, Brazil, September 2020 - February 2023. (DOCX)

## References

[1] Townsend JP, Hassler HB, Wang Z, Miura S, Singh J, Kumar S, et al. The durability of immunity against reinfection by SARS-CoV-2: a comparative evolutionary study. Lancet Microbe. 2021;2(12):e666–75. doi: 10.1016/S2666-5247(21)00219-6 34632431 PMC8486316

[2] O Murchu E, Byrne P, Carty PG, De Gascun C, Keogan M, O’Neill M, et al. Quantifying the risk of SARS-CoV-2 reinfection over time. Rev Med Virol. 2022;32(1):e2260. doi: 10.1002/rmv.2260 34043841 PMC8209951

[3] Cegolon L, Magnano G, Negro C, Larese Filon F, ORCHESTRA Working Group. SARS-CoV-2 Reinfections in Health-Care Workers, 1 March 2020-31 January 2023. Viruses. 2023;15(7):1551. doi: 10.3390/v15071551 37515237 PMC10384331

[4] Chemaitelly H, Ayoub HH, Tang P, Yassine HM, Al Thani AA, Hasan MR, et al. Addressing bias in the definition of SARS-CoV-2 reinfection: implications for underestimation. Front Med (Lausanne). 2024;11:1363045. doi: 10.3389/fmed.2024.1363045 38529118 PMC10961414

[5] ECDC. Reinfection with SARS-CoV-2: implementation of a surveillance case definition within the EU/EEA. European Centre for Disease Prevention and Control - Analysis and guidance. 2021 [cited 29 Aug 2023]. Available: https://www.ecdc.europa.eu/sites/default/files/documents/Reinfection-with-SARSCoV2-implementation-of-a-surveillance-case-definition.pdf

[6] Reino Unido. Investigation and management of suspected SARS-CoV-2 reinfections: a guide for clinicians and infection specialists. 15 Mar 2021 [cited 22 Jun 2025]. Available: https://www.gov.uk/government/publications/covid-19-investigation-and-management-of-suspected-sars-cov-2-reinfections/investigation-and-management-of-suspected-sars-cov-2-reinfections-a-guide-for-clinicians-and-infection-specialists

[7] Mensah AA, Lacy J, Stowe J, Seghezzo G, Sachdeva R, Simmons R, et al. Disease severity during SARS-COV-2 reinfection: a nationwide study. J Infect. 2022;84(4):542–50. doi: 10.1016/j.jinf.2022.01.012 35085659 PMC8786677

[8] Shahrbaf M, Alimohamadi Y, Yousefi Arfaei R, Salesi M, Izadi M, Raei M. Rate, risk factors, and clinical outcomes of SARS-CoV-2 reinfection vs. primary infection in readmitted COVID-19 patients in Iran: a retrospective cohort study. Front Public Health. 2024;12:1480805. doi: 10.3389/fpubh.2024.1480805 39484354 PMC11524883

[9] Rieke GJ, Monin MB, Breitschwerdt S, Boesecke C, Schlabe S. Confirmed SARS-CoV-2 Reinfection After 1 Year in a Patient with X-linked Agammaglobulinaemia. Infectious Diseases. 2022;1(1):35. doi: 10.17925/id.2022.1.1.35

[10] Nguyen NN, Nguyen YN, Hoang VT, Million M, Gautret P. SARS-CoV-2 Reinfection and Severity of the Disease: A Systematic Review and Meta-Analysis. Viruses. 2023;15(4):967. doi: 10.3390/v15040967 37112949 PMC10145185

[11] Choudhary MC, Crain CR, Qiu X, Hanage W, Li JZ. Severe Acute Respiratory Syndrome Coronavirus 2 (SARS-CoV-2) Sequence Characteristics of Coronavirus Disease 2019 (COVID-19) Persistence and Reinfection. Clin Infect Dis. 2022;74(2):237–45. doi: 10.1093/cid/ciab380 33906227 PMC8135388

[12] Bobrovitz N, Ware H, Ma X, Li Z, Hosseini R, Cao C, et al. Protective effectiveness of previous SARS-CoV-2 infection and hybrid immunity against the omicron variant and severe disease: a systematic review and meta-regression. Lancet Infect Dis. 2023;23(5):556–67. doi: 10.1016/S1473-3099(22)00801-5 36681084 PMC10014083

[13] Yamasaki L, Moi ML. Complexities in Case Definition of SARS-CoV-2 Reinfection: Clinical Evidence and Implications in COVID-19 Surveillance and Diagnosis. Pathogens. 2021;10(10):1262. doi: 10.3390/pathogens10101262 34684211 PMC8540172

[14] Ma KC, Dorabawila V, León TM, Henry H, Johnson AG, Rosenberg E, et al. Trends in Laboratory-Confirmed SARS-CoV-2 Reinfections and Associated Hospitalizations and Deaths Among Adults Aged ≥18 Years - 18 U.S. Jurisdictions, September 2021-December 2022. MMWR Morb Mortal Wkly Rep. 2023;72(25):683–9. doi: 10.15585/mmwr.mm7225a3 37347715 PMC10328471

[15] Pecoraro V, Pirotti T, Trenti T. Evidence of SARS-CoV-2 reinfection: analysis of 35,000 subjects and overview of systematic reviews. Clin Exp Med. 2023;23(4):1213–24. doi: 10.1007/s10238-022-00922-0 36289100 PMC9607758

[16] Soares R de CM, Mattos LR, Raposo LM. Risk Factors for Hospitalization and Mortality due to COVID-19 in Espírito Santo State, Brazil. Am J Trop Med Hyg. 2020;103(3):1184–90. doi: 10.4269/ajtmh.20-0483 32682453 PMC7470570

[17] Corona RA, Cunha AA da. COVID-19 in healthcare workers in the state of Espírito Santo, Brazil: clinical and sociodemographic characteristics associated with death and hospitalization. Einstein (Sao Paulo). 2022;20:eAO6241. doi: 10.31744/einstein_journal/2022AO6241 35293527 PMC8909153

[18] Garbin JRT, Leite FMC, Lopes-Júnior LC, Dell’Antonio CS da S, Dell’Antonio LS, Santos APBD. Analysis of Survival of Patients Hospitalized with COVID-19 in Espírito Santo, Brazil. Int J Environ Res Public Health. 2022;19(14):8709. doi: 10.3390/ijerph19148709 35886560 PMC9315540

[19] Dell’Antonio LS, Leite FMC, Dell’Antonio CS da S, Souza CB de, Garbin JRT, Santos APBD, et al. COVID-19 Mortality in Public Hospitals in a Brazilian State: An Analysis of the Three Waves of the Pandemic. Int J Environ Res Public Health. 2022;19(21):14077. doi: 10.3390/ijerph192114077 36360974 PMC9653571

[20] Brioschi Dos Santos AP, Vicente CR, Cola JP, Tanaka LF, Garbin JRT, Dell’Antonio LS, et al. The impact of COVID-19 on maternal death and fetal death, a cohort study in Brazil. PLoS One. 2023;18(8):e0290343. doi: 10.1371/journal.pone.0290343 37590217 PMC10434867

[21] Garbin JRT, Leite FMC, Dell’Antonio CSS, Dell’Antonio LS, Dos Santos APB, Lopes-Júnior LC. Hospitalizations for coronavirus disease 2019: an analysis of the occurrence waves. Sci Rep. 2024;14(1):5777. doi: 10.1038/s41598-024-56289-7 38459098 PMC10924092

[22] Pasha N, Akram M, Waqar N, Pasha K. Differential diagnosis of community-acquired pneumonia from Covid-19 by computed tomography. Biol Clin Sci Res J. 2023;2023(1):513. doi: 10.54112/bcsrj.v2023i1.513

[23] Pulliam JRC, van Schalkwyk C, Govender N, von Gottberg A, Cohen C, Groome MJ, et al. Increased risk of SARS-CoV-2 reinfection associated with emergence of Omicron in South Africa. Science. 2022;376(6593):eabn4947. doi: 10.1126/science.abn4947 35289632 PMC8995029

[24] Abedi P, Afshari P, Ansari S, Alavi SM, Dashtpayma S, Amiri H. The prevalence of and factors related to reinfection with COVID-19 in Ahvaz, Iran: A comparative cross-sectional study. Health Sci Rep. 2023;6(7):e1420. doi: 10.1002/hsr2.1420 37492272 PMC10363822

[25] Montes-González JA, Zaragoza-Jiménez CA, Antonio-Villa NE, Fermín-Martínez CA, Ramírez-García D, Vargas-Vázquez A, et al. Protection of hybrid immunity against SARS-CoV-2 reinfection and severe COVID-19 during periods of Omicron variant predominance in Mexico. Front Public Health. 2023;11:1146059. doi: 10.3389/fpubh.2023.1146059 37081954 PMC10110947

[26] Perez G, Banon T, Gazit S, Ben MS, Wortsman J, Grupel D, et al. A 1 to 1000 SARS-CoV-2 reinfection proportion in members of a large healthcare provider in Israel: a preliminary report. medRxiv. 2021; 2021.03.06.21253051. 10.1101/2021.03.06.21253051

[27] Deng L, Li P, Zhang X, Jiang Q, Turner D, Zhou C, et al. Risk of SARS-CoV-2 reinfection: a systematic review and meta-analysis. Sci Rep. 2022;12(1):20763. doi: 10.1038/s41598-022-24220-7 36456577 PMC9714387

[28] Flacco ME, Acuti Martellucci C, Baccolini V, De Vito C, Renzi E, Villari P, et al. Risk of reinfection and disease afterSARS‐CoV‐2 primary infection: Meta‐analysis. Eur J Clin Investigation. 2022;52(10). doi: 10.1111/eci.13845 PMC9353414 35904405

[29] Ismail NF, Rahman AE, Kulkarni D, Zhu F, Wang X, Del Carmen Morales G, et al. Incidence and outcome of SARS-CoV-2 reinfection in the pre-Omicron era: A global systematic review and meta-analysis. J Glob Health. 2023;13:06051. doi: 10.7189/jogh.13.06051 37994839 PMC10667793

[30] Chen Y, Zhu W, Han X, Chen M, Li X, Huang H, et al. How does the SARS-CoV-2 reinfection rate change over time? The global evidence from systematic review and meta-analysis. BMC Infect Dis. 2024;24(1). doi: 10.1186/s12879-024-09225-z PMC10956270 38515023

[31] Barbosa Costa W, Chagas de Assis S, Gurgel do Carmo N, Dias Mendoza A, Dos Santos da Silva R, Zarpelon LF, et al. Covid-19 E Sua Primeira Onda: Uma Análise Retrospectiva Dos Primeiros Casos Em Uma Cidade Fronteiriça Do Brasil. RECIMA21. 2023;4(2):e422767. doi: 10.47820/recima21.v4i2.2767

[32] Rizzatti M, Cezar Spode PL, Bouvier Erthal D, Faria RM de. Avaliação geográfica para risco de COVID-19 em população a partir de 50 anos na área urbana de Santa Maria, RS, Brasil. Geog Ens Pesq. 2020;:e10. doi: 10.5902/2236499444287

[33] Carvalho HFN de, Morais TR de, Albuquerque LP, Girão MMF, Silva MRL e, Silva CGL da, et al. Estudo dos dados epidemiológicos dos casos de Covid-19 na região metropolitana do Cariri, Ceará, Brasil. Braz J Hea Rev. 2022;5(6):22806–17. doi: 10.34119/bjhrv5n6-078

[34] ESPÍRITO SANTO. Portaria No 184-R, de 22 de setembro de 2020. Diário Oficial do Estado do Espírito Santo. Vitória; 2020. Available: https://saude.es.gov.br/Media/sesa/coronavirus/Portarias/PORTARIA%20184-R%20%20TESTAGEM%20CONTATOS.pdf http://ioes.dio.es.gov.br/portal/visualizacoes/html/4777/#e:4777

[35] Mukherjee A, Anand T, Agarwal A, Singh H, Chatterjee P, Narayan J, et al. SARS-CoV-2 re-infection: development of an epidemiological definition from India. Epidemiol Infect. 2021;149:e82. doi: 10.1017/S0950268821000662 33766185 PMC8027559

[36] Oliveira GP de, Bierrenbach AL de S, Camargo KR de, Coeli CM, Pinheiro RS. Accuracy of probabilistic and deterministic record linkage: the case of tuberculosis. Rev Saude Publica. 2016;50:49. doi: 10.1590/S1518-8787.2016050006327 27556963 PMC4988803

[37] Pacheco AG, Saraceni V, Tuboi SH, Moulton LH, Chaisson RE, Cavalcante SC, et al. Validation of a hierarchical deterministic record-linkage algorithm using data from 2 different cohorts of human immunodeficiency virus-infected persons and mortality databases in Brazil. Am J Epidemiol. 2008;168(11):1326–32. doi: 10.1093/aje/kwn249 18849301 PMC2638543

[38] Oliveira GP de, Bierrenbach AL de S, Camargo KR de, Coeli CM, Pinheiro RS. Accuracy of probabilistic and deterministic record linkage: the case of tuberculosis. Rev Saude Publica. 2016;50:49. doi: 10.1590/S1518-8787.2016050006327 27556963 PMC4988803

[39] Garcia KKS, Miranda CB de, Sousa FNEF de. Procedures for health data linkage: applications in health surveillance. Epidemiol Serv Saude. 2022;31(3):e20211272. doi: 10.1590/S2237-96222022000300004 36287481 PMC9887966

[40] Soares C, Dell’antonio S, Soares L, Antonio D’, Curto MR, Bierrenbach AL. Importância Da Qualidade E Definições Uniformes Para Os Dados De Vacinação Contra A Covid-19. Anais New Science Publishers | Editora Impacto. 2024 [cited 22 Jun 2025]. 10.56238/I-CIM-073

[41] ESPÍRITO SANTO. Nota Técnica COVID-19 75/2020. Notas Técnicas da Secretaria de Saúde do Estado do Espírito Santo. Vitória: Notas Técnicas Estaduais; 2020. Available: https://saude.es.gov.br/Media/sesa/coronavirus/Notas%20T%C3%A9cnicas/NOTA%20TECNICA%20COVID.19%20N.%2075.20%20%20Isolamento%20de%20casos,%20Rastreamento%20e%20Monitoramento%20de%20Contatos%20de%20Casos.pdf

[42] Cava F, San Román J, Barreiro P, Candel FJ, Álvarez-Timón FJ, Melero D, et al. Temporal Series Analysis of Population Cycle Threshold Counts as a Predictor of Surge in Cases and Hospitalizations during the SARS-CoV-2 Pandemic. Viruses. 2023;15(2):421. doi: 10.3390/v15020421 36851635 PMC9959442

[43] Campi G, Perali A, Marcelli A, Bianconi A. Sars-Cov2 world pandemic recurrent waves controlled by variants evolution and vaccination campaign. Sci Rep. 2022;12(1):18108. doi: 10.1038/s41598-022-22816-7 36302922 PMC9612611

[44] Bali Swain R, Lin X, Wallentin FY. COVID-19 pandemic waves: Identification and interpretation of global data. Heliyon. 2024;10(3):e25090. doi: 10.1016/j.heliyon.2024.e25090 38327425 PMC10847870

[45] Wolf JM, Kipper D, Borges GR, Streck AF, Lunge VR. Temporal spread and evolution of SARS-CoV-2 in the second pandemic wave in Brazil. J Med Virol. 2022;94(3):926–36. doi: 10.1002/jmv.27371 34596904 PMC8661965

[46] Hall VJ, Foulkes S, Charlett A, Atti A, Monk EJM, Simmons R, et al. SARS-CoV-2 infection rates of antibody-positive compared with antibody-negative health-care workers in England: a large, multicentre, prospective cohort study (SIREN). Lancet. 2021;397(10283):1459–69. doi: 10.1016/S0140-6736(21)00675-9 33844963 PMC8040523

[47] Fonseca PLC, Malta FSV, Braga-Paz I, do Prado Silva J, de Souza CSA, de Aguiar RS, et al. SARS-CoV-2 reinfection rate before and after VOC Omicron emergence: a retrospective study in Brazil. Braz J Microbiol. 2024;55(4):3959–64. doi: 10.1007/s42770-024-01467-y 39048913 PMC11711811

[48] Gómez-Gonzales W, Chihuantito-Abal LA, Gamarra-Bustillos C, Morón-Valenzuela J, Zavaleta-Oliver J, Gomez-Livias M, et al. Risk Factors Contributing to Reinfection by SARS-CoV-2: A Systematic Review. Adv Respir Med. 2023;91(6):560–70. doi: 10.3390/arm91060041 38131876 PMC10740414

[49] Galasso V, Pons V, Profeta P, Becher M, Brouard S, Foucault M. Gender differences in COVID-19 attitudes and behavior: Panel evidence from eight countries. Proc Natl Acad Sci U S A. 2020;117(44):27285–91. doi: 10.1073/pnas.2012520117 33060298 PMC7959517

[50] Abu-Raddad LJ, Chemaitelly H, Bertollini R, National Study Group for COVID-19 Epidemiology. Severity of SARS-CoV-2 Reinfections as Compared with Primary Infections. N Engl J Med. 2021;385(26):2487–9. doi: 10.1056/NEJMc2108120 34818474 PMC8631440

[51] La Torre G, Paglione G, Barone LC, Cammalleri V, Faticoni A, Marte M, et al. Evaluation of the Factors Associated with Reinfections towards SARS-CoV-2 Using a Case Control Design. J Clin Med. 2023;12(11):3861. doi: 10.3390/jcm12113861 37298055 PMC10253296

[52] Nonaka CKV, Gräf T, Barcia CA de L, Costa VF, de Oliveira JL, Passos R da H, et al. SARS-CoV-2 variant of concern P.1 (Gamma) infection in young and middle-aged patients admitted to the intensive care units of a single hospital in Salvador, Northeast Brazil, February 2021. Int J Infect Dis. 2021;111:47–54. doi: 10.1016/j.ijid.2021.08.003 34390857 PMC8356754

[53] Altarawneh HN, Chemaitelly H, Ayoub HH, Tang P, Hasan MR, Yassine HM, et al. Effects of previous infection, vaccination, and hybrid immunity against symptomatic Alpha, Beta, and Delta SARS-CoV-2 infections: an observational study. EBioMedicine. 2023;95:104734. doi: 10.1016/j.ebiom.2023.104734 37515986 PMC10404859

[54] Naveca FG, Nascimento VA, Nascimento F, Ogrzewalska M, Pauvolid-Corrêa A, Araújo MF, et al. SARS-CoV-2 intra-host diversity, antibody response, and disease severity after reinfection by the variant of concern Gamma in Brazil. Sci Rep. 2023;13(1):7306. doi: 10.1038/s41598-023-33443-1 37147348 PMC10160723

[55] Fralick M, Nott C, Moggridge J, Castellani L, Raudanskis A, Guttman DS, et al. Detection of COVID-19 Outbreaks in Long-Term Care Homes Using Built Environment Testing for SARS-CoV-2: A Multicentre Prospective Study. medRxiv. 2022; 2022.06.28.22276560. doi:10.1101/2022.06.28.22276560

[56] Slezak J, Bruxvoort K, Fischer H, Broder B, Ackerson B, Tartof S. Rate and severity of suspected SARS-Cov-2 reinfection in a cohort of PCR-positive COVID-19 patients. Clin Microbiol Infect. 2021;27(12):1860.e7-1860.e10. doi: 10.1016/j.cmi.2021.07.030 34419576 PMC8373524

[57] Ukwishaka J, Ndayishimiye Y, Destine E, Danwang C, Kirakoya-Samadoulougou F. Global prevalence of coronavirus disease 2019 reinfection: a systematic review and meta-analysis. BMC Public Health. 2023;23(1):778. doi: 10.1186/s12889-023-15626-7 37118717 PMC10140730

