# Supplementary material for: Epidemiological patterns of SARS-CoV-2 reinfections in Espírito Santo, Brazil: A population-based analysis using integrated surveillance and vaccination data
Source: PLoS One. 2025 Sep 10;20(9):e0331771. doi: 10.1371/journal.pone.0331771 (PMC12422470; doi:10.1371/journal.pone.0331771)
Supplement: S1 Table — (DOCX) [file pone.0331771.s001.docx]

Table S1. COVID-19 reinfection cases stratified by age group and vaccination status according to four methodological definitions, Espírito Santo, Brazil, September 2020 - February 2023.

A) Absolute distribution by methodology

| **Age Group** | **Vaccination Status** | **Type 1** | **Type 2** | **Type 3** | **Type 4** |
| --- | --- | --- | --- | --- | --- |
| **10-19 years** | 1st Dose | 4 | 18 | 52 | 126 |
|  | 2nd Dose | 24 | 106 | 315 | 783 |
|  | Unique | 0 | 2 | 4 | 6 |
|  | Additional Dose | 1 | 1 | 2 | 7 |
|  | One booster | 9 | 22 | 187 | 311 |
|  | More than one booster | 2 | 8 | 70 | 51 |
|  | Subtotal | 40 | 157 | 630 | 1,284 |
| **20-29 years** | 1st Dose | 23 | 114 | 247 | 732 |
|  | 2nd Dose | 190 | 664 | 2,039 | 4,912 |
|  | Unique | 0 | 1 | 14 | 38 |
|  | Additional Dose | 7 | 15 | 69 | 130 |
|  | One booster | 86 | 251 | 148 | 2,518 |
|  | More than one booster | 13 | 39 | 560 | 654 |
|  | Subtotal | 319 | 1,084 | 4,409 | 8,984 |
| **30-39 years** | 1st Dose | 21 | 110 | 171 | 596 |
|  | 2nd Dose | 210 | 835 | 2,067 | 5,339 |
|  | Unique | 13 | 54 | 160 | 443 |
|  | Additional Dose | 10 | 29 | 123 | 196 |
|  | One booster | 151 | 457 | 2,371 | 4,334 |
|  | More than one booster | 35 | 101 | 1,164 | 1,376 |
|  | Subtotal | 440 | 1,586 | 6,056 | 12,284 |
| **40-49 years** | 1st Dose | 12 | 55 | 121 | 349 |
|  | 2nd Dose | 114 | 528 | 1,512 | 4,264 |
|  | Unique | 6 | 31 | 78 | 244 |
|  | Additional Dose | 6 | 18 | 94 | 194 |
|  | One booster | 105 | 357 | 2,065 | 4,045 |
|  | More than one booster | 33 | 89 | 1,194 | 1,555 |
|  | Subtotal | 276 | 1,078 | 5,064 | 10,651 |
| **50-59 years** | 1st Dose | 2 | 34 | 61 | 194 |
|  | 2nd Dose | 79 | 346 | 809 | 2,456 |
|  | Unique | 0 | 6 | 12 | 43 |
|  | Additional Dose | 5 | 23 | 59 | 150 |
|  | One booster | 52 | 232 | 1,289 | 2,814 |
|  | More than one booster | 28 | 82 | 1,191 | 1,812 |
|  | Subtotal | 166 | 723 | 3,421 | 7,469 |
| **60+ years*** | 1st Dose | 1 | 19 | 20 | 102 |
|  | 2nd Dose | 14 | 117 | 214 | 732 |
|  | Unique | 0 | 0 | 6 | 13 |
|  | Additional Dose | 1 | 13 | 62 | 200 |
|  | One booster | 41 | 225 | 659 | 2,033 |
|  | More than one booster | 29 | 146 | 1,586 | 3,227 |
|  | Subtotal | 86 | 521 | 2,547 | 6,307 |
| **TOTAL** |  | 1,327 | 5,149 | 22,130 | 46,982 |

*Includes 60-69, 70-79, 80-89, and 90+ years age groups combined

B) Proportion of "More than one booster" by age group

| **Methodology** | **10-19 years** | **20-29 years** | **30-39 years** | **40-49 years** | **50-59 years** | **60+ years** |
| --- | --- | --- | --- | --- | --- | --- |
| **Type 1** | 5.0% | 4.1% | 8.0% | 12.0% | 16.9% | 33.7% |
| **Type 2** | 5.1% | 3.6% | 6.4% | 8.3% | 11.3% | 28.0% |
| **Type 3** | 11.1% | 12.7% | 19.2% | 23.6% | 34.8% | 62.3% |
| **Type 4** | 4.0% | 7.3% | 11.2% | 14.6% | 24.3% | 51.2% |

Legend:

Reinfection Type 1: Patients with two or more positive PCR tests separated by at least 90 days, with at least one intervening negative PCR result.

Reinfection Type 2: Patients with two consecutive positive PCR tests separated by at least 90 days, without an intervening negative result.

Reinfection Type 3: Patients with two or more positive results (PCR or TRa) separated by at least 90 days, with at least one intervening negative test (PCR or TRa).

Reinfection Type 4: Patients with two consecutive positive results (PCR or TRa) separated by at least 90 days, without requiring an intervening negative test.

*PCR: Polymerase Chain Reaction; TRa: Rapid Antigen Test*
